# The influence of balanced complex chromosomal rearrangements on preimplantation embryonic development potential and molecular karyotype

**DOI:** 10.1186/s12864-020-6731-9

**Published:** 2020-04-29

**Authors:** Gang Li, Weiyi Shi, Wenbin Niu, Jiawei Xu, Yihong Guo, Yingchun Su, Yingpu Sun

**Affiliations:** Reproductive Medical Center, First Affiliated Hospital of Zhengzhou University, No.1 Jianshe Road, Zhengzhou, China

**Keywords:** Balanced complex chromosome rearrangements, Preimplantation genetic testing, Assisted reproductive technology, Next-generation sequencing technology

## Abstract

**Background:**

Balanced complex chromosome rearrangements (BCCR) are balanced chromosomal structural aberrations that involve two or more chromosomes and at least three breakpoints. It is very rare in the population. The objective is to explore the difference of influence of three types of BCCR on early embryonic development and molecular karyotype.

**Results:**

Twelve couples were recruited including four couples of three-way rearrangements carriers (group A), three couples of double two-way translocations carriers (group B) and five couples of exceptional CCR carriers (group C). A total of 243 oocytes were retrievedin the seventeen preimplantation genetic testing (PGT) cycles, and 207 of these were available for fertilization. After intracytoplasmic sperm injection, 181oocytes normally fertilized. The rates of embryos forming on day3 in three groups were 87.88, 97.78 and77.14%, which was significantly different (*P* = 0.01). Compared with group B, the rate of embryo formation was statistically significantly lower in group C (*P* = 0.01). Furthermore, the rates of high-quality blastocysts in three group were 14.71, 48.15 and 62.96%, respectively, which was significantly different (*P* = 0.00). Compared with group B andC, the rate of high-quality blastocysts in group A was statistically significantly lower (*P* = 0.00;*P* = 0.00).

Comprehensive chromosome analysis was performed on 83 embryos, including 75 trophectodermcellsand 8 blastomeres. Except 7 embryos failed to amplify, 9.01%embryos were diagnosed as euploidy, and 90.91% were diagnosed as abnormal. As for group A, the euploid embryo rate was 10.71%and the abnormal embryo rate was 89.29%. In group B,the euploid embryo rate was 3.85%, the abnormal embryo rate was 96.15%. The euploid embryo rate was 13.04%, the abnormal embryo rate was 86.96% in group C. There were no significant differences among the three groups (*P* = 0.55).

**Conclusions:**

The lowest rate of high quality blastocysts has been for three-way rearrangements and the lowest rate of euploidy has been for double two-way translocations, although no significant difference. Different types of BCCR maybe have little effect on the embryonic molecular karyotype. The difference of influence of BCCR on early embryonic developmentandmolecular karyotypeshould be further studied.

## Background

Balanced complex chromosome rearrangements (BCCR) are balanced chromosomal structural aberrations that involve two or more chromosomes and at least three breakpoints. Most of them involve three or more chromosomes and three or more break points. It is very rare in the populationwith approximately 0.5% neonatal morbidity [1, 2]. BCCR carriers are rare but varied. According to the chromosome structure and complexity, BCCRs are usually classified into three types [3–5]: three-way rearrangements, double two-way translocations, and exceptional CCR. Three-way rearrangements is a kind of BCCR including three chromosome break points and the exchange of chromosome fragment. Double two-way translocationsrefers to two or three separate, simple reciprocal translocation or robertsoniantranslcation. Exceptional CCR is rearrangement of chromosomes, which has more than one breaking point, and the rearrangement level can be extremely complicated, often merging inversion and insert structure distortion. The first two involve translocations only, whereas exceptional CCR often includes other structural distortions, such as translocation, inversion, insertion, and others. Some CCR carriers often have growth deformities, mental retardation and congenital abnormalities [6–9]. Although many researchers have tried to explain the mechanism of CCR, it is still not clear.

However, most BCCR carriers have normal phenotypes, but they have a higher risk of reproductive failure. When one of the couples is a BCCR carrier, the possibility of producing normal gametes is theoretically much lower than that of carriers with single-chromosome abnormalities. Therefore, recurrent spontaneous abortion, arrested intrauterine pregnancy (aIUP), fetal malformation and infertility often happen. In addition, male BCCR can also be observed as oligoasthenoterazoospermia and infertile [7, 10]. It has been reported that the odds of balanced or normal embryos in couples with BCCR are < 6% [1]. Preimplantation genetic testing (PGT) is performed before embryo transfer, and a small portion of cells will be aspirated for comprehensive chromosome screening to analyze embryos identified as balanced or normal for transplantation. The method can reduce the miscarriage rate and improve clinical outcomes [11]. However, the odds of normal or balanced embryo for BCCR carriers is very low. The effects of different types of BCCR on embryonic development andmolecular karyotype are not clear.

In our study, clinical and laboratory data on preimplantation genetic testing to assist reproduction for three different types of BCCR carriers were collectedto explorethe difference of impacts of three types of BCCRs on embryo development and embryonic molecular karyotype.

## Results

### General conditions

Twelve BCCR couples diagnosed by karyotype analysis of peripheral blood were recruited including four female BCCR carriersand eight male BCCR carriers. 17 PGT cycles wereperformed. All twelve couples had negative reproductive history. Twelve BCCR couples were divided into three groups such as four couples of three-way rearrangements carriers (group A), three couples of double two-way translocations carriers (group B) and five couples of exceptional CCR carriers (group C) (Table 1). There were no significantly statistical differences among the three groups within the baseline information (Table 2).

**Table 1:** Chromosome karyotype of BCCRs couples

Groups	Case No.	Female’s karyotype	Male’s karyotype	Abnormal pregnancy history
A	1	46,XX	46,XY,t(1;16;4)(p22;q22;q23)	Arrested intrauterine pregnancy one time; artificial abortion one time; spontaneous abortion one time.
A	2	46,XX	46,XY,t(8;10;13)(q21;p12;q33)	Arrested intrauterine pregnancy one time.
A	3	46,XX,t(1;15;9)(q21;q11.2;q12)	46,XY	spontaneous abortion two times.
A	4	46,XX	46,XY,t(8;18;9)(q24.2;q21.2;cp22)	Arrested intrauterine pregnancy one time.
B	5	46,XX	46,XY,t(2;4)(q21;q31),t(2;5)(p23;q35)	Artificial abortion one time.
B	6	45,XX,t(6;13)(p21.1;q34)der(15;21)(q10;q10)	46,XY	spontaneous abortion four times.
B	7	46,XX,t(1;11)(q44;q23),t(2;8)(q31;p23)	46,XY	No pregnancy
C	8	46,XX,t(2;11)(q22;q24),inv.(13)(q12q32)	46,XY	Ectopic pregnancy one time.
C	9	46,XX	46,XY,t(1;11)(p10;p10),inv.(11)(q13q14)	Labor induction one time due to omphalocele.
C	10	46,XX	46,XY,t(1;8)(p22;p23)Ins(1;11)(p22;q23q25)	Biochemical pregnancy three times.
C	11	46,XX	45,XY,inv.(1)(p11q12),rob(15;22)(q10;q10)	No pregnancy
C	12	46,XX	45,XY,inv.(5)(p13q23),der(14;15)(q10;q10)	Arrested intrauterine pregnancy three times.

**Table 2:** Baseline information of the couples of BCCR carriers

	Group A	Group B	Group C	*P*
Famale age (years)	27.43 ± 2.70	27.33 ± 2.70	32.86 ± 5.70	0.14
Male age (years)	27.86 ± 2.672	30.33 ± 2.67	33.29 ± 4.19	0.15
BMI (kg/m^2^)^a^	20.39 ± 1.32	22.01 ± 4.06	21.29 ± 2.58	0.61
AFC(N)^b^	15.14 ± 7.88	15.33 ± 4.51	8.86 ± 3.98	0.14

*BMI* Body mass index, *AFC* Antral follicles count

### Controlled ovarian stimulation (COS) outcome of the PGT cycles for BCCR couples

After undergoing 17 cycles of COS in12 couples, a total of 243 oocytes were retrieved, including 207 mature oocytes (MII), and 181 (87.43%) oocytes were observed as two-pronuclear embryos (2PN) after intracytoplasmic sperm injection (ICSI). Then, 83 embryos were biopsied, including 8 embryos at the cleavage stage and 75 blastocysts. The rates of 2PN in group A, B and C were 69.47%(66/95), 83.33%(45/54) and 74.46%(70/94). And there was no significant differences among the three groups (χ^2^ = 3.48, *P* = 0.18, *P* > 0.05). The rates of embryos formation on day3 of three groups were 87.88%(58/66), 97.78%(44/45) and 77.14%(54/70), which was significantly different (χ^2^ = 10.05, *P* = 0.01, *P* < 0.05). Further analysis indicated that compared with group B, the rate of embryo formation was statistically significantly lower in group C (χ^2^ = 7.69, *P* = 0.01, *P* < 0.02). The rates of high-quality embryos on day3 in three group were 70.68%(41/58), 79.55%(35/44) and 75.93%(41/54) respectively, and there was no significant differences(χ^2^ = 1.08, *P* = 0.58, *P* > 0.05).

The rate of blastocyst formation on D5/6 were 51.52%(34/66), 60.00%(27/45) and 38.57%(27/70), which was also no significant differences(χ^2^ = 5.38, *P* = 0.07, *P* > 0.05). However, the rates of high-quality blastocysts in three group were 14.71%(5/34), 48.15%(13/27) and 62.96%(17/27), which was significantly different (χ^2^ = 15.77, *P* = 0.00, *P* < 0.05). Compared with groups B and C, the rate of high-quality blastocysts in group A was statistically significantly lower (χ^2^ = 8.09, *P* = 0, χ^2^ = 15.20, *P* = 0, *P* < 0.02) (Table 3).

**Table 3:** Effect of three types of BCCRs on embryo development in PGT cycles

Groups	retrieved oocytes	MII oocytes	The 2PN rate(n)	Day3 embryo formation rate(n)	the rate of high quality embryos at day3(n)	the rate of blastocyst formation(n)	the rate of high quality blastocyst(n)
A	95	84	69.47%(66/95)	87.88%(58/66)	70.68%(41/58)	51.52%(34/66)	14.71%(5/34)*
B	54	53	83.33%(45/54)	97.78%(44/45)	79.55%(35/44)	60.00%(27/45)	48.15%(13/27)
C	94	70	74.46%(70/94)	77.14%(54/70)*	75.93%(41/54)	38.57%(27/70)	62.96%(17/27)
Total	243	207	87.44%(181/207)	86.19%(156/181)	75.00%(117/156)	48.62%(88/181)	39.77%(35/88)

* *P* < 0.05 was considered statistically significant

### Embryo identification of the PGT cycles for BCCR couples

Eighty three embryos were biopsied, including 28 blastocysts in group A, 8 cleavage embryos and 22 blastocysts in group B and 25 blastocysts in group C. After comprehensive chromosome analysis, the total euploid embryo rate was 9.09% (7/77), the total aneuploidy rate was 90.91% (70/77), and 6 failed to amplify (Table S1).

Among them, the euploid embryo rate was 10.71%(3/28) and the aneupliodyrate was 89.29%(25/28) in group A. The euploid embryo rate was 3.85%(1/26), the aneupliody rate was 96.15%(25/26), and 4 embryos failed to diagnose in group B. The euploid embryo rate was 13.04%(3/23), theaneupliody rate was 86.96%(20/23), and 2 embryos failed to amplify in group C. There were no significant differences among the three groups (Fisher exact probabilities *P* = 0.55, *P* > 0.05).

**Fig. 1:**
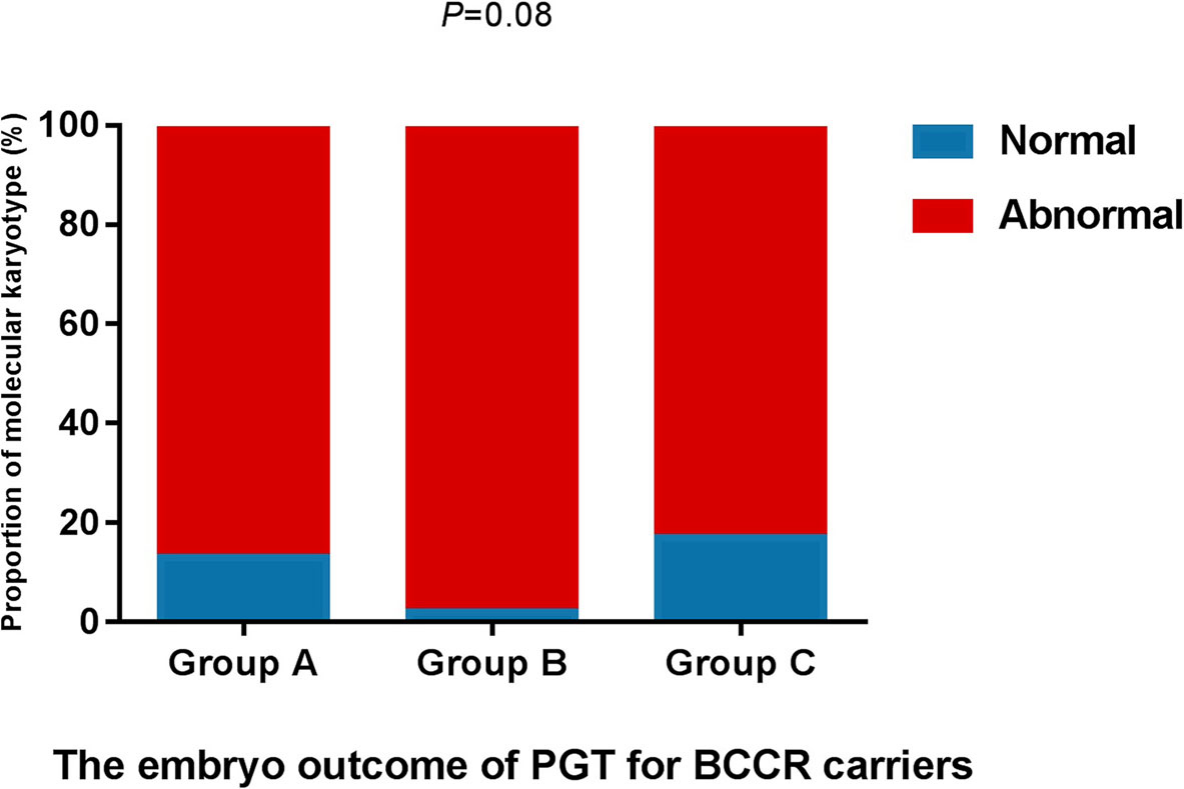
The embryo outcome of PGT for BCCR carriers. The balanced or normal embryo rate and abnormal embryo rate were 13.71%(17/124) and 86.29%(107/124) in the group of three-way rearrangements carriers (group A). The balanced or normal embryo rate was 2.5%(1/40) and the abnormal embryo rate was 97.5%(39/40) in the group of double two-way translocations (group B). The balanced or normal embryo rate was 17.07%(7/41) and the abnormal embryo rate was 82.93%(34/41) in the group of exceptional CCRs (group C). There was no significant difference in the embryonic molecular karyotypes among the three groups (Fisher exact probabilities *P* = 0.08, *P* > 0.05)

Due to the rarity of BCCR carriers, in order to increase sample size to explore the impact of BCCR on embryonic molccular karyotype, wecollecteddata from PGT for BCCR carriers reported in PubMed up to now. Frumkin T et al. reported a couple in which the husband was a three-way rearrangements carrier with one PGT cycle, who had 2 euploid embryos and 5 abnormal embryos [12]. Chan Tian et al. reported a couple in which the male partner was an exceptional CCR carrier with one PGT cycle, who had no euploid embryos and 2 aneuploid embryos [13]. E. Vanneste et al. reported a couple in which the husband was an exceptional CCRs carrier with two PGT cycles, who had 4 balanced or normal embryos and 12 abnormal embryos [14]. Paul et al. reported 4 couples, including 3 males and 1 female three-way rearrangements carriers with 6 PGT cycles, who had 6 euploid embryos and 31 aneuploid embryos [10]. Hu L et al.reported 7 couples, including 5 couples with three-way rearrangements, who had 3 balanced or normal embryos, 31 abnormal embryos and one embryo of amplification failure, and 1 couple with double two-way translocations, who had no balanced or normal embryos and 12 abnormal embryos [1]. Brunet BCFK et al. reported 3 couples with three-way rearrangements with 4 cycles, who had 3 balanced or normal embryos and 15 abnormal embryos, and 1 double two-way translocations who had no balanced or normal embryos and 2 abnormal embryos in one cycle [11]. Therefore, in summary, the euploid embryo rate and aneuploidy rate were 13.71%(17/124) and 86.29%(107/124) ingroup A. The euploid embryo rate was 2.5%(1/40) and the aneuploidy rate was 97.5%(39/40) in group B. Theeuploid embryo rate was 17.07%(7/41) and the aneuploidy rate was 82.93%(34/41) in group C, respectively. (Fig. 1). There was no significant difference in the embryonic molecular karyotypes among the three groups (Fisher exact probabilities *P* = 0.08, *P* > 0.05).

### Clinical outcome of PGT cycles for BCCR couples

**Fig. 2:**
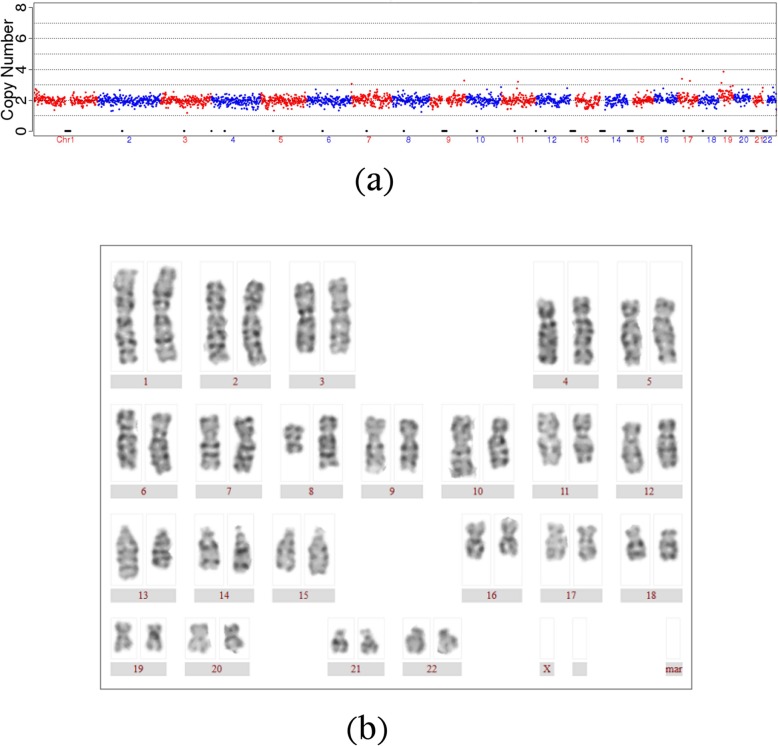
The NGS-PGT outcome and prenatal diagnosis of the normal delivery of Case 2. **a** The NGS-PGT outcome was euploidy. **b** The prenatal diagnosis at 16 weeks of gestation was 46, XN, t (8,13,10) (q21;q31;p15). (XN means XX or XY)

In the 17 PGT cycles, there were 13 cycles in which no euploid embryo could be transplanted and 4 cycles in which euploid embryos were transplanted with frozen-thawed embryo transfer. Two of the 4 cycles was clinically pregnant, and the prenatal diagnosis at 16 weeks of gestation was 46, XN and 46, XN, t (8,13,10)(q21;q31;p15), (XN means XX or XY). The outcome mentioned that euploid embryos of NGS-PGT for BCCR maybe the balanced translocation carriers. (Fig. 2) Fortunately, two boys were born alive and healthy.

## Discussion

BCCR carriers have a high risk of recurrent spontaneous abortion and giving birth to offspring with abnormal karyotypes [15, 16]. In this study, although a couple (Case No. 9) had a natural pregnancy with a healthy girl, othereleven couples had negative reproductive history, including spontaneous abortions, artificial abortions, arrested intrauterine pregnancy (aIUP) and odinopoeia. Thus, BCCR carriers are advised to maintain contraception, and natural pregnancy should be carefully considered. If natural pregnancy occurs, regular perinatal care and prenatal assessment is needed.

A study showed that embryos carrying unbalanced chromosomal translocations undergo delayed development and asynchronous cleavage that may lead to implantation failure or miscarriage [17]. But the difference of the influence of BCCR on early embryonic development is unclear. Our data showed that, compared with double two-way rearrangement carriers, there was a significant decline in the embryo formation rate on Day3 after fertilization in exceptional CCRs carriers. Compared with double two-way rearrangement and exceptional CCRs, three-way rearrangements had a significantly lower rate of high-quality blastocysts. However, there was no significant difference in the fertilization rate, the embryo formation rate on Day3, and blastocyst formation rate among the three groups, suggesting that three types of BCCRs may have some differenteffects on high-quality blastocyst formation but not on embryos formation on Day3. A study showed that compared with exceptional CCRs, there was no significant difference in three-way rearrangements in the high-quality embryo rate on D3 after fertilization, while the former was significantly lower than the latter, which was inconsistent with our results [1]. Another study suggested some three-way complex translocations and complex CCR result in poor embryonic development, and were found to have more mosaicism. But we only observed one mosaicism in case.2. Therefore, the effect of BCCR on early embryonic development should be further studied with larger samples to draw a more accurate conclusion.

Our data show that the odds of obtaining a euploid embryo are 9.01%. The effect of three types of BCCR on the embryonic molecular karyotype was analyzed and the results present that exceptional CCRs had more balanced or normal embryos, but the difference between the three groups was not statistically significant. After Analysis with the data reported in the literature, it showed that exceptional CCRs had more balanced or normal embryos, but the difference between the three groups was stillnot statistically significant and the sample size needs to be increased in the future.

The application of PGT for BCCR carriers is controversial. Some scholars believedthat PGT is an effective fertility treatment for BCCR carriers, while others opposedit [1, 18, 19]. Twelve BCCR couples were treated with PGT, and two couples succeeded in getting pregnantand delivered healthy babies. Although the odds of having a balanced or normal embryo are low, PGT reduces the risk of miscarriage and is the only way to help BCCR carriers give birth to related offspring. In addition, 33.73%(28/83) abnormal embryos were new chromosome abnormalities. Therefore, it was necessary to use comprehensive chromosome analysis to perform genetic testing.

## Conclusions

The lowest rate of high quality blastocysts has been for three-way rearrangements and the lowest rate of euploidy has been for double two-way translocations, although no significant difference. Different types of BCCR had little effect on the embryonic molecular karyotype. Although it’s difficult to get normal embryos for BCCR carriers, PGT can reduce the risk of miscarriage and is the only way to help BCCR couple give birth to related offspring. Due to the limited sample, the effects of different BCCR types on early embryo development and embryo molecular karyotype need to be studied further by expanding the sample size.

## Methods

### Study patients

TwelveBCCR couples underwent 17 cycles of PGT in the Reproductive MedicineCenter of the First Affiliated Hospital of Zhengzhou University from May 2011 to June 2019 and clinical and laboratory datawas collected and analyzed retrospectively. All study methods were approved by Institutional Review Board and Ethics Committee of the First Affiliated Hospital of Zhengzhou University, and were performed in accordance with relevant guidelines and regulations. All subjects enrolled in the study gave written formal consent to participate.

### PGT procedure

The case.12 was conducted with long-acting GnRH agonist long protocol in follicular phase and the other eleven female patients were treated with a long luteal phase stimulation protocol for controlled ovarian stimulation (COS). Oocytes were observed closely after ICSI, and all embryos were transferred into equilibrated medium and cultured at 37°Cin a CO_2_ incubator. The embryos were scored on Day3 and Day5/6 according to the criterion thatembryos on Day3 that scored ≥6 C-II were considered high-quality embryos, andblastocysts on Day5/6 that scored ≥3BB were considered high-quality blastocysts.

Case.8 performed blastomeres biopsied on Day3, and the others were biopsied at blastocyst stage on Day5/6. Two to five cells were biopsied, and comprehensive gene amplification was performed. Next, these biopsied cells were assessed by single nucleotide polymorphism microarray (SNP microarray) (HumanCytoSNP-12, Illumina company, resolution about 5-10 M) or next generation sequencing (NGS) (Hiseq2500, Illumina company, resolution about 4 M) technology. Then, vitrification was performed, and embryos identified as euploidy were subjected to frozen-thawed embryo transfer.

### Statistical methods

Baseline materials were analyzed by one-way ANOVA. The rates were compared by chi-square test and Fisher exact probabilities, and *P* < 0.05 was considered statistically significant. Pairwise comparisons among three groups were corrected by Bonferroni post hoc tests, and *P* < 0.02 was considered statistically significant.

## Supplementary information

**Additional file 1: Table S1.** The outcome of SNP or NGS.

## Data Availability

The datasets analysed during the current study are available in the link: https://pan.baidu.com/s/10HULNPhZsimdPZAGpOOopw with code i27w.

## Abbreviations

BCCR: Balanced complex chromosome rearrangements
ICSI: Intracytoplasmic sperm injection
COS: Controlled ovarian stimulation
MII: Mature oocytes
PGT: Preimplantation genetic testing
SNP: Single nucleotide polymorphism
NGS: Next generation sequencing
