# Supplementary material for: The influence of balanced complex chromosomal rearrangements on preimplantation embryonic development potential and molecular karyotype
Source: BMC Genomics. 2020 Apr 29;21:326. doi: 10.1186/s12864-020-6731-9 (PMC7191696; doi:10.1186/s12864-020-6731-9)
Supplement: Supplementary file 1 — Additional file 1: Table S1. The outcome of SNP or NGS. [file 12864_2020_6731_MOESM1_ESM.doc]

Table S1 The outcome of SNP or NGS

| Group | Case NO. | NO. | NGS/SNP array | Embryo kynotype |
| --- | --- | --- | --- | --- |
| A | 1 | 1 | NGS | 46,XY, +16q(q21→qter,~18M,×3) |
| A | 1 | 2 | NGS | 46,XX |
| A | 2 | 3 | NGS | 46,XY,-8q(q11.23→q13.3,~20M,×1,),+10p(pter→p13,~16M,×3),-13q(q21.1→qter,~56M,×1) |
| A | 2 | 4 | NGS | 47,XY,-8q(q12.1→qter,~82M,×1),+10p(pter→p13,~15M,×3),+16(×3) |
| A | 2 | 5 | NGS | 46,XY,-11(×1,mos,~70%) |
| A | 2 | 6 | NGS | 46,XY,-8q(q12.1→q13.2,~11M,×1),+10p(pter→p13,~15M,×3),+10q(q23.1→qter,~50M,×3),-13q(q21.33→qter,~45M,×1) |
| A | 2 | 7 | NGS | 47,XY,+8q(q12.1→qter,~85M,×3),+10(×3) |
| A | 2 | 8 | NGS | 46,XY |
| A | 2 | 9 | NGS | 46,XX |
| A | 3 | 10 | NGS | Multiple chromosome abnormality |
| A | 3 | 11 | NGS | 46,XX,+1p(pter→p35.2,~28M,×3),+1(p36.13→q21.1,~129M,×4),-9p(pter→p13.2,~38M,×1),+9q(q21.11→qter,~68M,×3) |
| A | 3 | 12 | NGS | 48,XX,+1p(pter→p12,~117M,×3),-9p(pter→p13.2,~38M,×1),+20(×3),+21(×3) |
| A | 3 | 13 | NGS | 46,XY,-1p(pter→p11.2,~120M,×1),+5(p13.3→q14.1,~49M,×3),+15(pter→q21.1,~23M,×3) |
| A | 4 | 14 | NGS | Amplification failure |
| A | 4 | 15 | NGS | 43,XO,-8(×1),-9(×1) |
| A | 4 | 16 | NGS | Multiple chromosome abnormality |
| A | 4 | 17 | NGS | 46,XY,-8q(q24.22→q24.3,~14M,×1),+9p(pter→p21.3,~21M,×3) |
| A | 4 | 18 | NGS | 46,XY,-9p(pter→p21.2,~27M,×1),-18(pter→q21.1,~47M,×1) |
| A | 4 | 19 | NGS | Multiple chromosome abnormality |
| A | 4 | 20 | NGS | 46,XX,+9(p21.2→qter,~113M,×3),-18(pter→q21.1,~47M,×1) |
| A | 4 | 21 | NGS | 46,XX,+8q(q24.22→qter,~14M,×3),-18q(q21.1→qter,~30M,×1) |
| A | 4 | 22 | NGS | 46,XX,+9(p21.2→qter,~113M,×3),-18(pter→q21.1,~47M,×1) |
| A | 4 | 23 | NGS | Multiple chromosome abnormality |
| A | 4 | 24 | NGS | 45,XX,+8q(q24.22→qter,~14M,×3),-9p(pter→p21.1,~30M,×1),-19(×1) |
| A | 4 | 25 | NGS | 46,XX,-8q(q23.3→qter,~32M,×1),+9p(pter→p21.2,~26M,×3),+9(p21.1→q22.1,~59M,×3),-18p(pter→p11.21,~14M,×1) |
| A | 4 | 26 | NGS | 46,XX,+5(q33.3→q34,~5M,×3),+9(p21.1→q21.13,~43M,×3) |
| A | 4 | 27 | NGS | 46,XY,+8q(q24.22→q24.3,~14M,×3),-9p(pter→p21.1,~31M,×1) |
| A | 4 | 28 | NGS | Multiple chromosome abnormality |
| B | 5 | 29 | SNP array | 46,XX |
| B | 5 | 30 | SNP array | 47, XX, +22 |
| B | 5 | 31 | SNP array | Amplification failure |
| B | 5 | 32 | SNP array | 46, XY, dup(2)(q21→qter) |
| B | 5 | 33 | SNP array | 46, XX,dup(5)(q35→qter) |
| B | 5 | 34 | SNP array | Amplification failure |
| B | 5 | 35 | SNP array | 46, XY, dup(2)(q21.1→qter), dup(5)(q35→qter) |
| B | 5 | 36 | SNP array | Amplification failure |
| B | 6 | 37 | NGS | 47,XX,+16(×3) |
| B | 6 | 38 | NGS | 47,XXY,+13q(q31.1→qter,~27M,×3) |
| B | 6 | 39 | NGS | 46,XX,-6(p12.3→qter,~119M,×1),-6q(q12→q13,~11M,×1),+13(pter→q33.1,~83M,×3) |
| B | 6 | 40 | NGS | 46,XY,-4p(pter→p13,~40M,×1),+6p(pter→p21.1,~42M,×3),-13q(q33.1→qter,~11M,×1),-15q(q24.3→qter,~24M,×1),-16p(pter→p12.3,~20M,×1),-17(pter→q12,~34M,×1) |
| B | 6 | 41 | NGS | 45,XY,-4(pter→q31.21,~142M,×1),+6p(pter→p12.3,~50M,×3),-13q(q33.1→qter,~11M,×1),-21(×1) |
| B | 6 | 42 | NGS | 44,XY,-6p(pter→p12.3,~50M,×1),+13(pter→q33.1,~84M,×3),+13q(q32.3→qter,~13M,×4),-14(×1),-21(×1) |
| B | 6 | 43 | NGS | 45,XX,+1(×3),-4q(q31.3→qter,~28M,×1),-6(p12.3→qter,~119M,×1),-6q(q22.31→q22.33,~9M,×1),-10(p11.21→q22.3,~46M,×1),+13(pter→q33.1,~82M,×3) |
| B | 6 | 44 | NGS | 46,XY,+3p(pter→p22.1,~43M,×3),-20p(pter→p12.3,~8M,×1) |
| B | 6 | 45 | NGS | 46,XX,+3p(p21.1→p12.1,~31M,×3),+3q(q25.1→q27.3,~34M,×3),+6q(q14.3→qter,~84M,×3),+7p(p15.3→p12.3,~24M,×3) |
| B | 6 | 46 | NGS | 47,XY,+15(pter→q23,~41M,×4),+15(pter→q15.2,~10M,×4),+15q(q21.3→q25.2,~27M,×4),+15q(q24.1→qter,~26M,×4),+21(×3) |
| B | 6 | 47 | NGS | 46,XY,-4(×1),+5(p13.3→qter,~150M,×3),-6p(pter→p12.3,~50M,×1),+11(×3),+12(pter→q13.12,~50M,×3),-12q(q13.12→qter,~82M,×1) |
| B | 6 | 48 | NGS | 46,XX,-6p(pter→p12.3,~50M,×1) |
| B | 6 | 49 | NGS | 46,XX,-1p(p31.2→p21.1,~38M,×1),+2q(q12.1→q24.3,~61M,×3),+2q(q32.3→q36.3,~33M,×3),+3p(p14.2→p12.1,~25M,×3),-4(p15.2→q12,~33M,×1),-4q(q24→q28.1,~24M,×1),+5p(pter→p13.1,~38M,×3),+5q(q12.3→qter,~114M,×3),-6(p21.1→qter,~116M,×1),+7q(q22.2→qter,~54M,×3),+9(pter→q21.32,~85M,×3),-11p(p15.1→p12,~23M,×1),- |
| B | 7 | 50 | NGS | 46,XY,+2q(q32.1→qter,~53M,×3),-11q(q22.3→qter,~31M,×1) |
| B | 7 | 51 | NGS | 46,XY,-1q(q43→qter,~9M,×1),+2q(q31.2→qter,~61M,×3),+11q(q22.1→qter,~35M,×5) |
| B | 7 | 52 | NGS | Amplification failure |
| B | 7 | 53 | NGS | 46,XX,+2q(q24.3→qter,~70M,×3),-4q(q21.3→q28.3,~52M,×1),+6(p22.3→q16.3,~78M,×3),-11(pter→q12.3,~55M,×1),-11q(q21→qter,~37M,×1) |
| B | 7 | 54 | NGS | 46,XX,-2q(q32.1→qter,~57M,×1) |
| B | 7 | 55 | NGS | 46,XX,-11q(q14.2→qter,~47M,×1) |
| B | 7 | 56 | NGS | 46,XY,-2q(q31.1→qter,~65M,×1) |
| B | 7 | 57 | NGS | 46,XX,+2q(q31.3→qter,~59M,×3) |
| B | 7 | 58 | NGS | 46,XX,-2q(q32.1→qter,~57M,×1),-11q(q14.2→qter,~46M,×1) |
| C | 8 | 59 | SNP array | 46,XX |
| C | 8 | 60 | SNP array | 45,XY,-3 |
| C | 8 | 61 | SNP array | 46,XY,del(2)(q21.3→qter),dup(11)(q23.3→qter) |
| C | 8 | 62 | SNP array | 46,XY,dup(2)(q21.3→qter),del(11) (q23.3→qter) |
| C | 8 | 63 | NGS | 45,XY,-2q(q21.1→qter,~109M,×1),-22(×1) |
| C | 8 | 64 | NGS | 46,XX,-2p(pter→p16.1) |
| C | 8 | 65 | NGS | 45,XY,+2q(q21.2→qter,~104M,×3),-11q(q24.1→qter,~12M,×1),-17(×1) |
| C | 9 | 66 | NGS | 45,XX，-1q(q21.3→qter,~96M,×1),+11q(q21→qter,~38M,×3),-21(×1) |
| C | 9 | 67 | NGS | 46,XX,+3(p24.1→q23,~112M,×3),+4q(q32.2→qter,~27M,×3),-5(pter→q13.2,~69M,×1),+6(p22.3→q16.3,~82M,×3),+6q(q21→qter,~62M,×3),+7p(p15.3→p11.2,~34M,×3),-10(×1),+11q(q22.3→qter,~29M,×3),+14q(q24.1→qter,~33M,×3),+16(p11.2→qter,~59M,×3),+22(×3) |
| C | 9 | 68 | NGS | 46,XY,-1(p35.3→q21.3,~123M,×1),+1q(q21.3→q31.1,~34M,×3),+1q(q31.3→q43,~44M,×3),-11q(q21→qter,~39M,×1) |
| C | 9 | 69 | NGS | 46,XX,+1q(q21.3→qter,~96M,×3),-11q(q21→qter,~39M,×1) |
| C | 9 | 70 | NGS | 47,XY,+1q(q21.3→qter,~96M,×3),-11q(q21→qter,~40M,×1),+16(×3) |
| C | 9 | 71 | NGS | 45,XX,-7(×1) |
| C | 10 | 72 | NGS | Multiple chromosome abnormality |
| C | 10 | 73 | NGS | 45,XY,-8(×1) |
| C | 10 | 74 | NGS | 46,XX,+11q(q22.3→q24.1,~20M,×3) |
| C | 10 | 75 | NGS | 46,XX,-11q(q22.3→qter,~25M,×1) |
| C | 11 | 76 | NGS | 48,XX,+9(×3),+13(×3) |
| C | 12 | 77 | SNP array | 47，XY，+16 |
| C | 12 | 78 | SNP array | 47，XY，+15 |
| C | 12 | 79 | SNP array | 46，XY,del(5)(q34→qter) |
| C | 12 | 80 | SNP array | 46,XN |
| C | 12 | 81 | SNP array | Amplification failure |
| C | 12 | 82 | SNP array | 46,XN |
| C | 12 | 83 | SNP array | Amplification failure |
